# Tartary Buckwheat (*Fagopyrum tataricum*) FtTT8 Inhibits Anthocyanin Biosynthesis and Promotes Proanthocyanidin Biosynthesis

**DOI:** 10.3390/ijms242417368

**Published:** 2023-12-11

**Authors:** Jiao Deng, Lijuan Wang, Lan Zhang, Chaojie Yang, Juan Huang, Liwei Zhu, Qingfu Chen, Ziye Meng, Fang Cai, Taoxiong Shi

**Affiliations:** School of Life Sciences, Research Center of Buckwheat Industry Technology, Guizhou Normal University, Guiyang 550025, China; ddj613@163.com (J.D.); ljw58273@163.com (L.W.); zhangl8720@163.com (L.Z.); 15685965728m@sina.cn (C.Y.); huang200669@163.com (J.H.); liweib0401001@163.com (L.Z.); cqf1966@163.com (Q.C.); iorimouse@126.com (Z.M.); kimico84@foxmail.com (F.C.)

**Keywords:** Tartary buckwheat, anthocyanins, proanthocyadinins, regulation mechanism

## Abstract

Tartary buckwheat (*Fagopyrum tataricum*) is an important plant, utilized for both medicine and food. It has become a current research hotspot due to its rich content of flavonoids, which are beneficial for human health. Anthocyanins (ATs) and proanthocyanidins (PAs) are the two main kinds of flavonoid compounds in Tartary buckwheat, which participate in the pigmentation of some tissue as well as rendering resistance to many biotic and abiotic stresses. Additionally, Tartary buckwheat anthocyanins and PAs have many health benefits for humans and the plant itself. However, little is known about the regulation mechanism of the biosynthesis of anthocyanin and PA in Tartary buckwheat. In the present study, a bHLH transcription factor (TF) FtTT8 was characterized to be homologous with AtTT8 and phylogenetically close to bHLH proteins from other plant species. Subcellular location and yeast two-hybrid assays suggested that FtTT8 locates in the nucleus and plays a role as a transcription factor. Complementation analysis in *Arabidopsis tt8* mutant showed that FtTT8 could not recover anthocyanin deficiency but could promote PAs accumulation. Overexpression of *FtTT8* in red-flowering tobacco showed that *FtTT8* inhibits anthocyanin biosynthesis and accelerates proanthocyanidin biosynthesis. QRT-PCR and yeast one-hybrid assay revealed that FtTT8 might bind to the promoter of *NtUFGT* and suppress its expression, while binding to the promoter of *NtLAR* and upregulating its expression in K326 tobacco. This displayed the bidirectional regulating function of FtTT8 that negatively regulates anthocyanin biosynthesis and positively regulates proanthocyanidin biosynthesis. The results provide new insights on TT8 in Tartary buckwheat, which is inconsistent with TT8 from other plant species, and FtTT8 might be a high-quality gene resource for Tartary buckwheat breeding.

## 1. Introduction

Tartary buckwheat (*Fagopyrum tataricum*), belonging to the genus *Fagopyrum* within the family Polygonaceae, is a kind of crop utilized for both food and medicine [1]. As the country of origin and main producer of Tartary buckwheat, China owns abundant germplasm resources and has a long cultivation history [2,3]. Moreover, Chinese people take full advantage of buckwheat to make a variety of food and health products, such as noodles, steamed bun, cakes, cookies, bread, pasta, tea, liquor, and pillows [4,5,6]. Buckwheat products attract more and more consumers worldwide due to their rich nutrition and human-beneficial metabolites, especially abundant bioactive flavonoids, like rutin, quercetin, tannin, and proanthocyanidin, among others [7,8]. Rutin has been reported to have numerous bioactive properties, including antioxidant, antihypertension, anti-inflammatory, antidiabetic, and gastric lesion protecting activities, as well as preventing cognitive impairments like Alzheimer’s disease [9,10,11].

Tartary buckwheat seeds have been utilized as rice and flour. However, their sprouts are also rich in nutrients, and possess the same health benefits as the seeds. Therefore, sprouts have become increasingly popular as functional greens worldwide [12,13]. Additionally, Tartary buckwheat sprouts contain no allergic proteins that exist in the seeds and contain anthocyanins that are absent in the seeds [14,15,16]. Anthocyanin is a kind of water-soluble natural pigment that belongs to the flavonoid family. Except for pigmentation for the flowers, fruits, leaves, etc., of plants, anthocyanin also plays roles in resistance to biotic and abiotic stress as well as in seed pollination and dispersal [17,18,19].

The anthocyanin biosynthesis pathway is one branch of the flavonoid biosynthesis pathway, which includes chalcone synthase (CHS), chalcone isomerase (CHI), flavanone 3-hydroxylase (F3H), dihydroflavonol-4-reductase (DFR), anthocyanidin synthase (ANS), and anthocyanin 3-*O*-glucosyltransferase (UFGT). In this pathway, two substrates, leucoanthocyantin and anthocyanidin, are catalyzed by leucoanthocyanin reductase (LAR) and anthocyanidin reductase (ANR), respectively, which leads to the biosynthesis of proanthocyanidins (PAs), another kind of flavonoid and contributes to the pigmentation of the plant’s seed coat [20,21,22]. Nowadays, the biosynthesis pathway and regulation mechanism of anthocyanin and PA have been well established and deeply characterized in model plants *Arabidopsis thaliana* [23,24,25], Petunia hybrida [26,27], *Zay maize* [28,29], as well as other plant species. Notably, three transcription factors, (TFs) MYB, bHLH, and WD40, are the main transcriptional regulators for flavonoid biosynthesis [30]. MYB and bHLH can play the role alone by binding to target gene promoters, while WD40 does not and it regulates flavonoid biosynthesis by forming a protein complex with MYB and bHLH (MBW) [30,31,32]. At present, some MYB transcription factors have been reported to be involved in the regulation of anthocyanin and proanthocyanidin biosynthesis [33,34,35,36,37]. FtMYB15 [33] promotes anthocyanins accumulation. The overexpression of FtMYB1 and FtMYB2 significantly enhances PAs accumulation in *Nicotiana tabacum* [34]. FtMYB3 [35], FtMYB8 [36], and FtMYB18 [37] act as negative regulators of anthocyanin and proanthocyanidin biosynthesis in Tartary buckwheat. However, the literature focuses on MYB, and the regulation functions of bHLH and WD40 remain under-researched. Here, a bHLH transcription factor gene *FtTT8* was characterized and verified to possess bidirectional regulation functions that inhibit anthocyanin accumulation and enhance proanthocyanidin biosynthesis. This conclusion is distinct from that of other plant species and provides new insights into TT8’s role in the regulation of flavonoid biosynthesis.

## 2. Results

### 2.1. Cloning and Molecular Characterization of FtTT8

**Figure 1:**
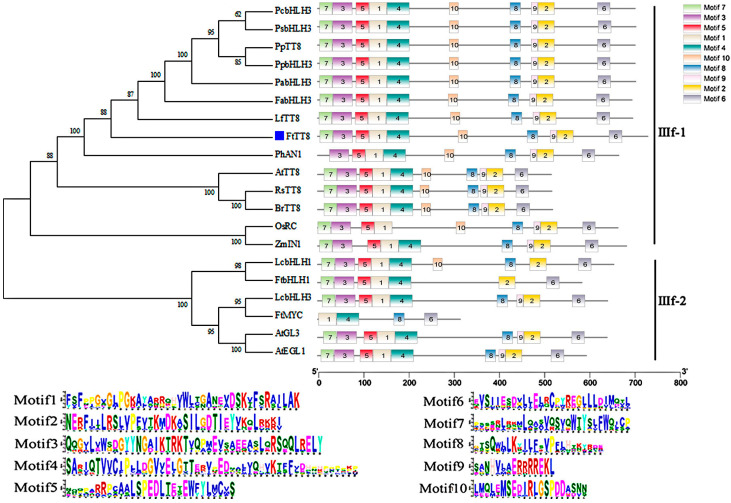
Phylogenetic tree and conserved motif analysis of the FtTT8 and bHLH transcription factors regulating anthocyanins and PAs in other species. Note: PcbHLH3 (Pc: *Prunus cerasifera*, ID: AKV89647.1), PpTT8 (Pp: *Prunus persica*, ID: XP_007200710.2), PpbHLH3 (ID: AIE57508.1), PhAN1 (Ph, *Petunia x hybrid*, ID: AAG25928), FabHLH3 (Fa, *Fragaria x ananassa*, ID: USN18571.1), LfTT8 (Lf: *Liquidambar formosana*, ID: QVX18577.1), PsbHLH3 (Fs, *Prunus salicina*, ID: XU25495.1), PabHLH3 (Pa, *Prunus avium*, ID: AJB28481.11), AtTT8 (At, *Arabidopsis thaliana*, ID: NP192720.2), AtGL3 (ID: AF246291), AtEGL1 (ID: AF027732), RsTT8 (Rs: *Raphanus sativus*, ID: ASF79354.1), BrTT8 (Br, *Brassica rapa subsp. Rapa*, ID: AEA03281), OsRC (Os, *Oryza sativa*, ABB17166.1), ZmIN1 (Zm, *Zea mays*, ID: AAB03841.1), LcbHLH1 (Lc, *Litchi chinensis*, ID: APP94122.1), LcbHLH3 (ID: APP94124.1), FtbHLH1 (Ft, *Fagopyrum tataricum*, ID: KT737454), and FtMYC (ID: KU162971).

**Figure 2:**
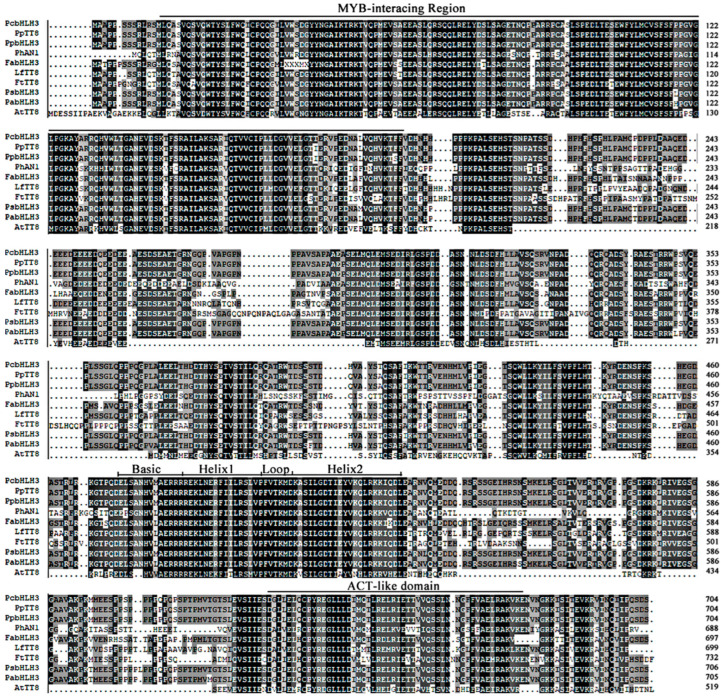
Protein sequence alignment of FtTT8 and other bHLH proteins that regulate anthocyanin and proanthocyanidin synthesis. Note: All nine species are the same as those listed in Figure 1.

The full length of the CDS sequence of *FtTT8* was obtained from Tartary buckwheat seedlings, which contained 2199 bp and encoded a protein of 732 amino acids. Sequence alignments with the NCBI database revealed FtTT8 to be a member of the bHLH transcription factor family. Phylogenetic analysis of FtTT8 and bHLH transcript factors from other species that have been reported to be involved in the regulation of anthocyanin and proanthocyanidin biosynthesis showed that the FtTT8 gene is closely related to LfTT8 and belongs to the IIIf subfamily (Figure 1). Most of these bHLH TFs contain 10 motifs (Figure 1). Since the LfTT8 TF was reported to regulate anthocyanin biosynthesis in *Liquidambar formosana* [38], we therefore speculated that the *FtTT8* gene is a strong candidate to be responsible for anthocyanin pigmentation in Tartary buckwheat. bHLH TFs from nine species that were more closely related to FtTT8 were chosen to analyze the conserved domains. The results showed that they contain a specific conserved bHLH (basic helix1 loop helix2) domain, the N-terminal MIR (MYB-interacting region), and the C-terminal ACT-like domain (Figure 2). The MIR is critical for binding to MYB TFs [39], therefore, FtTT8 may play a role in regulating anthocyanin or proanthocyanidin biosynthesis by interacting with the MYB protein.

### 2.2. Analysis of Subcellular Location and Transcription Activation of FtTT8

**Figure 3:**
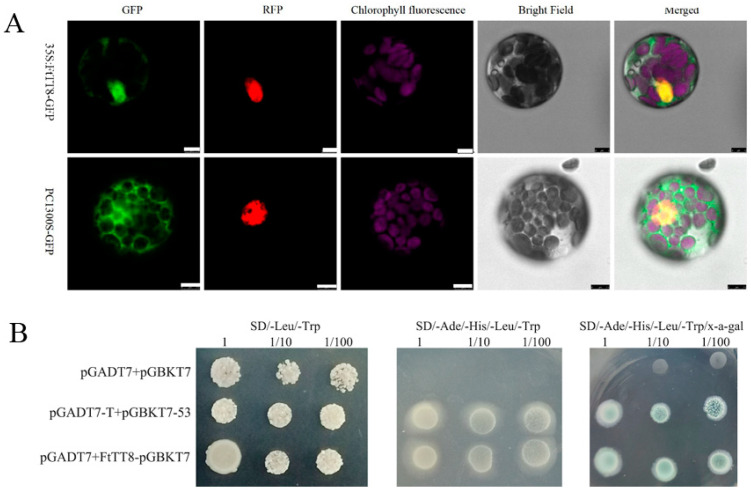
(**A**) Subcellular localization of the FtTT8 protein (scale bars = 7.5 μm). (**B**) Yeast two-hybrid assay, pGADT7 + pGBKT7 and pGADT7-T + pGBKT7-p53 were used as negative and positive controls, respectively.

Subcellular location analysis displayed that FtTT8-GFP merged with RFP (the nucleus location reference), while the GFP from the control vector was distributed throughout the cell (Figure 3A). This implied that FtTT8 is located in the nucleus and plays the role of a transcript factor. The result of the yeast two-hybrid assay suggests that the transcript factor FtTT8 possesses transcription activation activity (Figure 3B).

### 2.3. Complementation of the Arabidopsis tt8 Mutant by FtTT8

**Figure 4:**
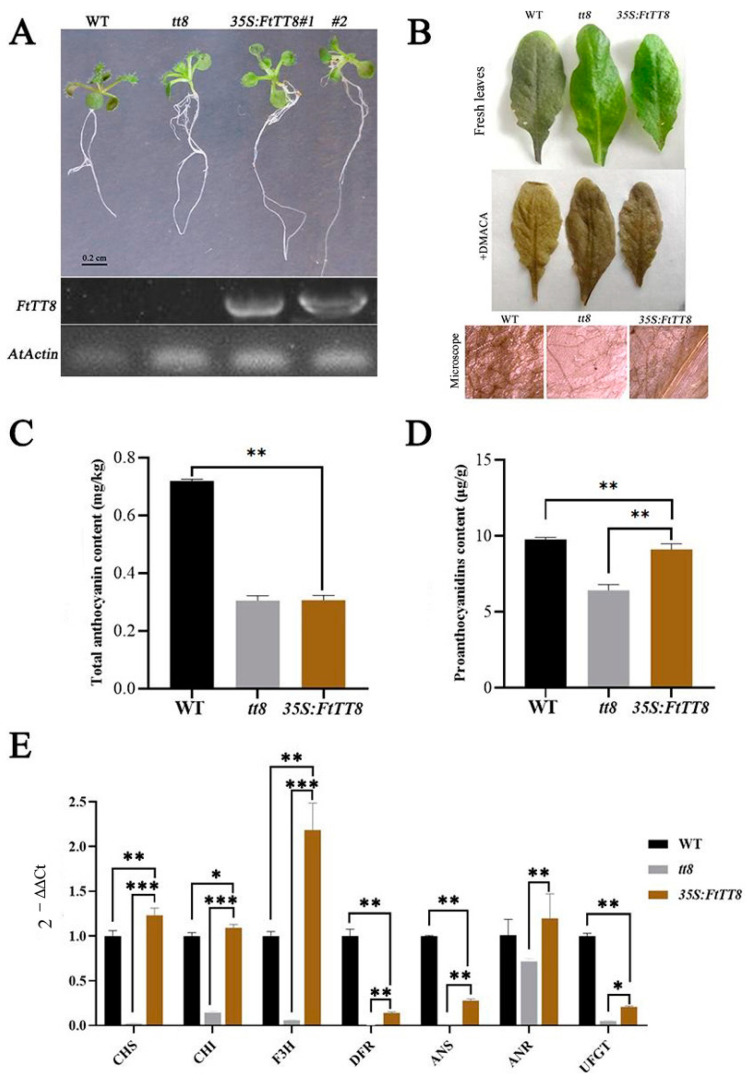
Complementation analysis of the *Arabidopsis tt8* mutant seedlings with FtTT8. (**A**) Seedlings of the WT, *attt8* mutant, and *FtTT8*-transformed plants and expression of FtTT8 in three types of *Arabidopsis* plants. (**B**) DMACA staining of leaves of the WT, *attt8* mutant, and *FtTT8* overexpressing Arabidopsis plants. (**C**) Total anthocyanin content. (**D**) PA content. (**E**) Expression changes of the anthocyanin and PA biosynthesis pathway genes by the overexpression of *FtTT8* in *Arabidopsis* leaves (* *p* < 0.05, ** *p* < 0.01, *** *p* < 0.001).

Complementation analysis in the *Arabidopsis attt8* mutant with *FtTT8* driven by the CaMV35S promoter was conducted to verify the function of FtTT8 in the regulation of anthocyanin and PA biosynthesis. FtTT8 transgenic *Arabidopsis* plants (three of each) were screened out and they showed a much higher expression level of *FtTT8*, whereas the wild-type (WT) and *attt8* mutant had no expression of this gene (Figure 4A). Firstly, when observing the seedlings of these tree types, the color red could obviously be observed in the leaves and hypocotyl of WT seedlings, while no red was evident in the *attt8* mutant and the *FtTT8* over expression lines (Figure 4A), indicating that *FtTT8* was unable to promote anthocyanin biosynthesis. Meanwhile, DMACA staining was performed for PA analysis. The results showed that the *attt8* mutant contained less PA than the WT; however, the PA content of the *attt8* mutant transformed by *FtTT8* increased remarkably, nearly to the WT plant level (Figure 4B). Content measurement of the total anthocyanins and PAs showed that the WT contained higher anthocyanins, while the *attt8* mutant and the *FtTT8*-transformed plants had similar anthocyanin content, and both were low (Figure 4C); the *attt8* mutant had lower PA content than the WT, but PA content was improved in the *FtTT8*-transformed plants, and close to the WT (Figure 4D), which was consistent with phenotype investigation.

To analyze the effect of FtTT8 on the expression of key genes in the anthocyanin and PA biosynthesis pathway, the expression levels of *CHS*, *CHI*, *F3H*, *DFR*, *ANS*, *UFGT*, and *ANR* in the leaves of the WT, *attt8* mutant, and *FtTT8*-transformed plants were analyzed by qRT-PCR. In the WT plants, except for *ANR*, the expression levels of other genes were very low, in the *FtTT8*-overexpression plants, most genes were up regulated, especially *CHS*, *CHI*, *F3H*, and *ANR*, which had higher expressions than those in the WT plants (Figure 4E). Key genes shared in the anthocyanin and PA biosynthesis pathway *DFR* and *ANS* increased slightly, while *UFGT* expression remained very low, not overly different from that of the mutant, but the gene in the key and speed-limiting enzyme ANR, which leads to the PA biosynthesis pathway, was significantly upregulated in the *FtTT8-* overexpression lines compared to the *attt8* mutant, and even higher than in the WT plant (Figure 4E).

**Figure 5:**
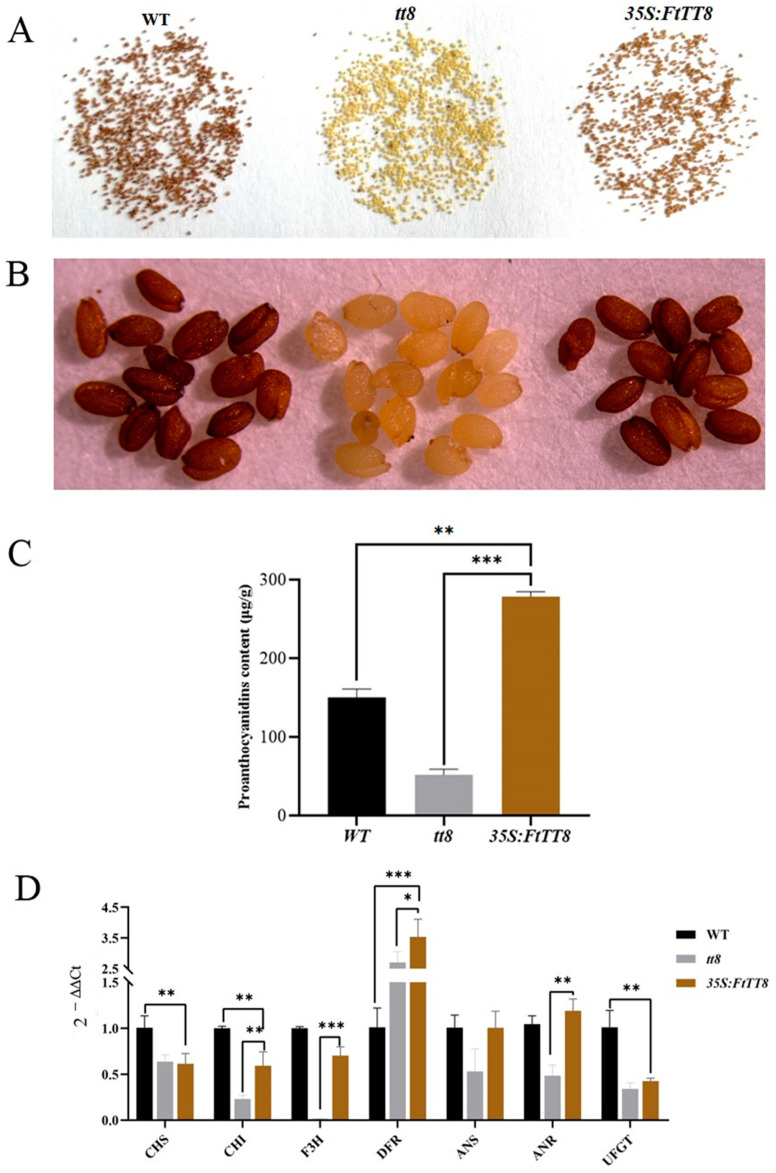
Complementation analysis of *Arabidopsis tt8* mutant seeds with *FtTT8*. (**A**) Seeds of the WT, *attt8* mutant, *FtTT8*-transformed *Arabidopsis* plants. (**B**) DMACA staining of the seeds of the WT, *attt8* mutant, and *FtTT8* overexpressing *Arabidopsis* plants. (**C**) PA content. (**D**) Expression changes of anthocyanin and PA biosynthesis pathway genes by the overexpression of *FtTT8* in *Arabidopsis* leaves (* *p* < 0.05, ** *p* < 0.01, *** *p* < 0.001).

The color of the WT seed coat was brown, while that of the *att8* mutant was light yellow. However, after the *attt8* mutant was complemented by *FtTT8*, the seed coat color returned to brown (Figure 5A). Consistently, DMACA staining showed that *attt8* mutant seed coats nearly had no PAs, while the seed coat color of the WT and transgenic plants changed to dark brown (Figure 5B), suggesting that they contained high levels of PAs content, and this was verified by the measurement of PA content (Figure 5C). Additionally, most common genes shared in the anthocyanin and PA biosynthesis pathway were upregulated in the *FtTT8*-transformed seeds (Figure 5D), whereas, UFGT was still lowly expressed and had no significant difference from that of the *attt8* mutant; however, *ANR* expression increased to even higher levels than those of the WT plants (Figure 5D). All the above results suggest that *FtTT8* could not accelerate anthocyanin accumulation but promotes PA biosynthesis in *Arabidopsis*. 

### 2.4. Overexpression of FtTT8 in K326 Tobacco

**Figure 6:**
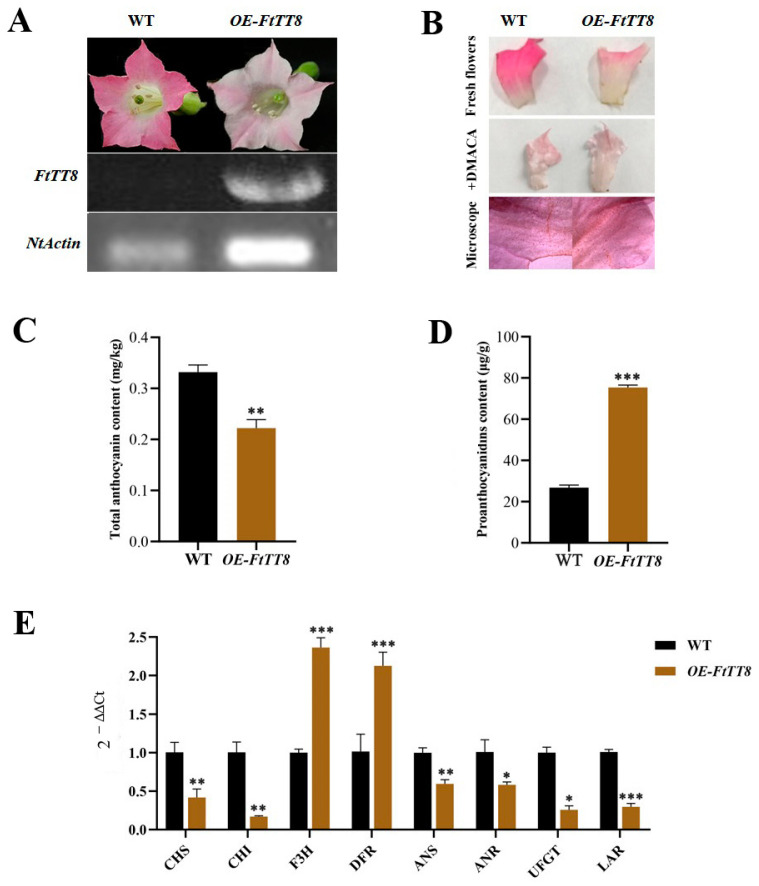
Overexpression analysis of *FtTT8* in the K326 tobacco flower. (**A**) Flowers of WT and *FtTT8*-transformed *K326 tobacco* (OE, overexpression). (**B**) DMACA staining of flowers of WT and *FtTT8* overexpressing tobacco. (**C**) Total anthocyanin content and (**D**) PA content in the WT and *FtTT8* overexpressing tobacco flowers. (**E**) Expression changes of the anthocyanin and PA biosynthesis pathway genes by the overexpression of *FtTT8* in tobacco flowers (* *p* < 0.05, ** *p* < 0.01, *** *p* < 0.001).

To further identify the function of FtTT8 in the regulation of anthocyanin and PA biosynthesis, overexpression of *FtTT8* in K326 tobacco (with red flowers) was conducted. The flower of wild-type K326 tobacco had no expression of the *FtTT8* gene, and its color was dark red; however, the *FtTT8*-transformed tobacco flower became light pink with high expression levels of *FtTT8* (Figure 6A). After DMACA staining, the brown color had no obvious difference between the WT and transgenic plants, and brown spots were similarly detected under the microscope (Figure 6B). Consistent with the results, the total anthocyanin content of the transgenic tobacco flower was significantly lower than that in theWT flower (Figure 6C), and the PA content showed no obvious difference between them (Figure 6D). The effect of FtTT8 on the expression profiles of structural genes in the anthocyanin and PA biosynthesis pathway were analyzed by qRT-PCR. Except for F3H and DFR, which were more highly expressed in the transgenic tobacco flower, the other genes were downregulated in the transgenic tobacco flower (Figure 6E).

**Figure 7:**
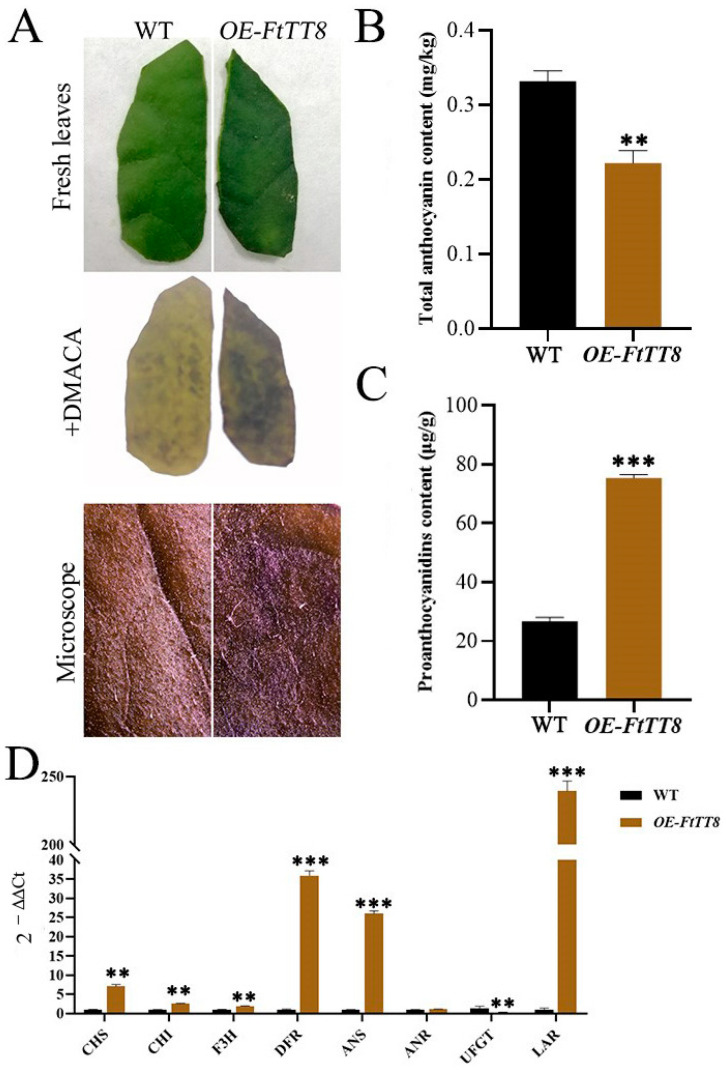
Overexpression analysis of *FtTT8* in K326 tobacco leaves. (**A**) DMACA staininf flowers of WT and *FtTT8* overexpressing tobacco. (**B**) Total anthocyanins content and (**C**) PAs content in WT and *FtTT8* overexpressing tobacco leaves. (**D**) Expression changes of anthocyanin and PA biosynthesis pathway genes by the overexpression of *FtTT8* in tobacco leaves (** *p* < 0.01, *** *p* < 0.001).

Interestingly, PAs accumulated more in the *FtTT8*-transformed tobacco leaves than in WT plants, but anthocyanin pigment was invisible to the naked eye (Figure 7A). Detecting the content of anthocyanins and PAs showed that total anthocyanins accumulated less while PAs accumulated more (about a three-fold difference) in transgenic tobacco leaves by *FtTT8* than those in WT plants (Figure 7B,C). When analyzing the expression levels of structural genes in the anthocyanin and PA biosynthesis pathway, common genes, including *CHS*, *CHI* and *F3H*, were slightly upregulated in the *FtTT8*-transformed tobacco leaves, while *DFR* and *ANS* were greatly increased (Figure 7D). However, *UFGT*, which catalyzed anthocyanin production, was downregulated in the transgenic plants, which was consistent with the lower content of total anthocyanin. However, surprisingly, in the two key genes, *ANR* and *LAR*, that lead to the branch of PA synthesis, the former saw no change, while the latter showed a dramatic approximately 250-fold increase in the transgenic plants (Figure 7D), indicating that *LAR* plays the main role in the catalyzation of PA production. These gene expression profiles were consistent with the pigment accumulation. All of the results suggest that FtTT8 negatively regulates anthocyanin biosynthesis but positively regulates PA biosynthesis when transformed in K326 tobacco.

### 2.5. FtTT8 Combined to the Promoters of NtUFGT and NtLAR

**Figure 8:**
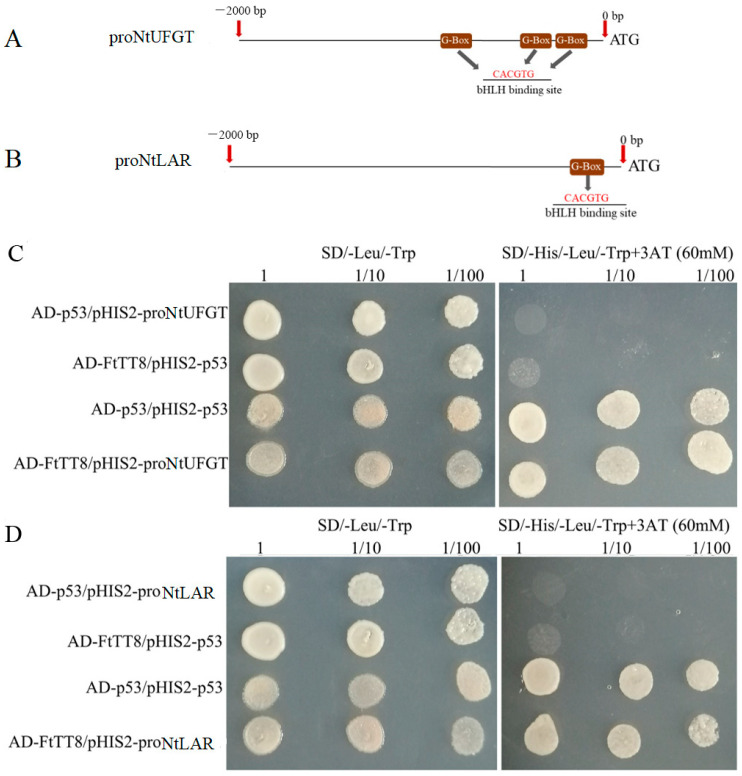
Analysis of the promoters of *NtUFGT* and *NtLAR* and yeast one-hybrid assays. G-box in the promoter of *NtUFGT* (**A**) and *NtLAR* (**B**), yeast one-hybrid assays for the combination between the FtTT8 and *NtUFGT* promoters (**C**), and FtTT8 and *NtLAR* (**D**), respectively.

UFGT and LAR were the speed-limiting enzymes that led to the anthocyanin synthesis branch and PA synthesis branch, respectively, in K326 tobacco, and the results of the qRT-PCR (Figure 6E and Figure 7D) show that *NtUFGT* displayed significantly different expression in the flowers and leaves of the WT and transgenic plants, and *NtLAR* expressed remarkable differently between the WT and transgenic tobacco leaves. Furthermore, both the 2000 bp promoters of *NtUFGT* and *NtLAR* contain G-box (CACGTG) (Figure 8A,B), which is reported to be a bHLH binding site [40]. Therefore, we speculate that *NtUFGT* and *NtLAR* might be the downstream target genes of the transcription factor FtTT8. To verify this hypothesis, a yeast one-hybrid experiment was performed, and the results suggested that FtTT8 could combine with the promoters of *NtUFGT* (Figure 8C) and *NtLAR* (Figure 8D), respectively.

## 3. Discussion

### 3.1. The Different Functions of FtTT8 in the Regulation Anthocyanin and Proanthocyanidin Biosynthesis

It has been well documented that AtTT8 positively regulates anthocyanin and PA biosynthesis in *Arabidopsis* [41], and that its homologs in other plant species, such as pear [42,43], lotus [44], strawberry [45], and *Medicago truncatula* [46], among others, have similar functions. For example, our previous study showed that in *NnTT8*-overexpression *Arabidopsis,* lotus NnTT8 can interact with AtMYB114 to promote anthocyanin and PA accumulation in leaves and seeds, which may be due to activating transcript levels of *ANS* and *UFGT* genes [44]. However, some bHLH TFs, including BnbHLH92a in *Brassica napus* [47] and bHLH92 in sheepgrass [48], inhibited anthocyanin and PA accumulation. Hu et al. reported that BnbHLH92a binds directly to the *ANS* gene promoter and represses its expression. In addition, BnbHLH92a can interact with a WD40 protein (BnTTG1) and represses the biosynthesis of anthocyanins and PAs in rapeseed [47]. Overexpression of LcbHLH92 in *Arabidopsis* significantly inhibited the expression levels of the *DFR* and *ANS* genes in leaves and seeds, and led to a decrease in anthocyanins and proanthocyanidins, respectively [48]. Phylogenetic relationship analysis showed that FtTT8 was closely related to LfTT8 and PhAN1 (Figure 1), all of them belonging to the IIIf-1 subfamily of bHLH TFs. Furthermore, like other bHLH regulators of anthocyanin and PA synthesis, the FtTT8 protein also contained a bHLH domain, a MYB-interacting region (MIR) and ACT-like domain (Figure 2). In addition, FtTT8 was located in the nucleus, and had transcriptional activation activity (Figure 3). These results indicate that FtTT8 was a transcription factor which may have a similar function to the other IIIf-1 subfamily of bHLHs in regulating anthocyanin and PA biosynthesis.

To verify this assumption, the complementation analysis of the *Arabidopsis tt8* mutant with *FtTT8* was conducted. The results were not consistent with the predicted results as there was no accumulation of anthocyanin pigments observed in the transgenic plant, where the color was just close to the colorless *attt8* mutant (Figure 4A). Additionally, the low anthocyanin content and no significant change in the expression of the anthocyanin-related gene *AtUFGT*, which encodes the speed-limiting enzyme (Figure 4C,E), indicated that FtTT8 cannot enhance anthocyanin accumulation in *Arabidopsis*. Conversely, the seed coat color of the *attt8* mutant was recovered by transformation with *FtTTT8* from light yellow to brown (Figure 5A), and DMACA staining (Figure 4B and Figure 5B), measurement of proanthocyanidin content (Figure 4D and Figure 5C) of *Arabidopsis* leaves and seeds, as well as the expression level of the speed-limiting anthocyanin-related genes *AtANR* (Figure 4B and Figure 5D), all suggested that *FtTT8* was able to promote proanthocyanidin biosynthesis.

The transformation of K326 tobacco (with red flowers) with *FtTT8* was conducted to further prove the functions of FtTT8. To our surprise, the flower color of the transgenic tobacco turned lighter than that of the WT (Figure 6A), and lower contents of anthocyanins in the flowers and leaves of the transgenic plant (Figure 6C and Figure 7B), as well as the downregulated expression level of the speed-limiting gene of anthocyanin biosynthesis *NtUFGT* (Figure 6E and Figure 7D), indicated that *FtTT8* prevented anthocyanin biosynthesis in tobacco. Although PA accumulation in the *FtTT8*-transformed tobacco showed no significant change in the flowers compared with the WT (Figure 6B,D), deeper DMACA staining (Figure 7A), a large increase in PA content (Figure 7C), and remarkably upregulated expression of *NtLAR* (Figure 7D) in the leaves of transgenic tobacco, demonstrated that *FtTT8* promoted PA accumulation in tobacco leaves.

Therefore, unlike other bHLH TFs, which either simultaneously improved anthocyanin and PA biosynthesis [44,46], or simultaneously suppressed anthocyanin and PA accumulation [47,48], *FtTT8* possessed bidirectional regulating effects on anthocyanin and PA biosynthesis that negatively regulated anthocyanin biosynthesis and positively regulated PA biosynthesis. This finding is new and interesting; however, the mechanism of different roles of FtTT8 on regulation of anthocyanin and PA biosyntheis pathways is unknown, it may be due to several different amino acid residues in the conserved domains in FtTT8 or for other reasons. The mechanism of competitiveness between the synthesis of both secondary metabolites remains unclear. Furthermore, if FtTT8 regulates other flavonoid composition syntheses, such as flavones, or flavonols, what regulates FtTT8 as well as which MYB and WD40 TFs can combine with it to play this function, need to be studied.

### 3.2. FtTT8 Interacted with the Promoter of NtUFGT and NtLAR to Play Roles in the Regulation of Anthocyanin and Proanthocyanidin Biosynthesis

**Figure 9:**
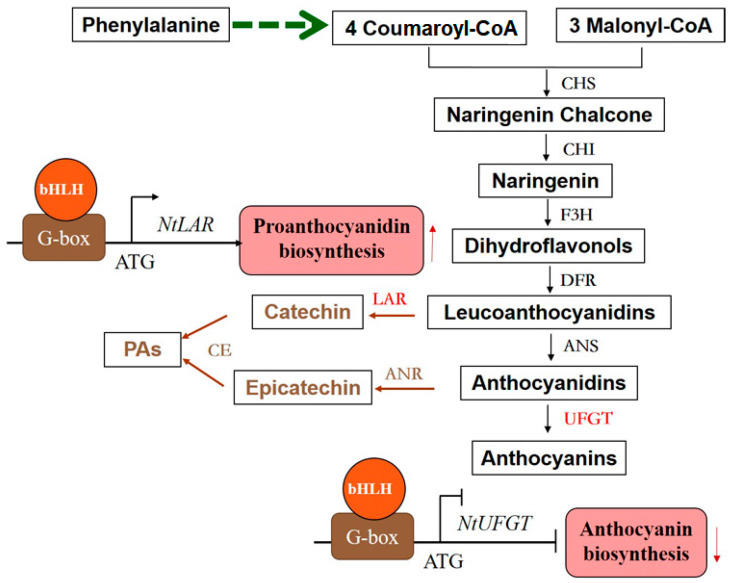
Anthocyanin and PA biosynthesis pathways and FtTT8 regulation in K326 tobacco. Note: ↑ means positive regulation and ↓ means negative regulation.

It has been reported that bHLH TFs play roles by interaction with G-box DNA element of their regulated gene in most plants [40,49]. For example, AtbHLH106 confers salt tolerance on *Arabidopsis* by integrating the functions of multiple genes through its G-box [50]. NtUFGT and NtLAR were the speed-limiting enzymes, which lead to the anthocyanin and proanthocyanidin synthesis branch, respectively in tobacco (Figure 9). Combined with the expression level obtained by qRT-PCR, that showed downregulation of *NtUFGT* (Figure 6E) and upregulation of *NtLAR* (Figure 7D), which was consistent with anthocyanin (Figure 6A,C) and PA accumulation (Figure 7A,C), both *NtUFGT* and *NtLAR* promoters contained G-box (Figure 8A,B), indicating that *NtUFGT* and *NtLAR* were probably the downstream target genes of FtTT8. Therefore, we speculate that FtTT8 interacts with the G-box of the *NtUFGT* promoter, and represses the expression of *NtUFGT*, resulting in less anthocyanin biosynthesis in transgenic tobacco flowers and leaves. Meanwhile, FtTT8 interacts with the G-box of the *NtLAR* promoter, and activates the expression of *NtLAR*, leading to improved proanthocyanidins accumulation in transgenic tobacco leaves (Figure 9).

## 4. Materials and Methods

### 4.1. Plant Materials

The seeds of Tartary buckwheat cultivar ‘Jinqiao 2’ from the Research Center of Buckwheat Industry Technology, Guizhou Normal University were planted based on the paper bed germination method with some modification [51], and placed in an artificial climate box at 25 °C, under a 16 h/8 h photoperiod and 80% humidity. The seven-day sprouts were collected and frozen in liquid N_2_ immediately and stored at −80 °C for further experiments.

### 4.2. Cloning and Characterization of FtTT8

The protein of Tartary buckwheat TT8 (FtTT8) was obtained by homologous alignment with *Arabidopsis* AtTT8 (AT4G09820) based on a Tartary buckwheat database (http://mbkbase.org/Pinku1/ (accessed on 8 August 2023)), and full-length specific primers were designed using Primes 5 according to the ORF sequence of the *FtTT8* gene. The primer sequences are listed in Table S1.

Extraction of the total RNA of the samples mentioned above was performed using an OminiPlant RNA kit (Kangwei, Beijing, China), and cDNA was synthesized using a ReverTra Ace^®^ qPCR RT kit (Toyobo, Osaka, Japan) according to the manufacturer’s instructions. Using the cDNA as the template, and full-length specific primers obtained above, the CDS of *FtTT8* was amplified by a PCR program using high-fidelity thermostable DNA polymerase (TaKaRa, Dalian, China). Then, the amplified PCR product was cloned to T vector (pMD19-T, TaKaRa, China), and transformed to *E.coil* strain DH5α and the detected positive clones were sent to a biological company (Sangon Biotech, Shanghai, China) for sequencing. Phylogenic analysis was achieved using MEGA 7.026 software [52], and motif prediction was analyzed by MEME (https://meme-suite.org/meme/tools/meme (accessed on 12 August 2023)), then visualized using the TBtools program [53]. bHLH transcription factors from other species involved in the regulation of anthocyanin and PA biosynthesis were obtained from the NCBI database by sequence alignments with FtTT8. Multi-alignment was performed using the online website for multiple sequence alignment by CLUSTALW (https://www.genome.jp/tools-bin/clustalw (accessed on 13 August 2023)).

### 4.3. Arabidopsis Protoplast Transient Expression Assay

The CDS of *FtTT8* was amplified and cloned into the pC1300S-GFP vector using the homologous recombination method, and the specific primers are listed in Table S1. The recombined vector pC1300S-GFP-FtTT8 and reference vector Ghd7:RFP (located in the nucleus) were co-transformed into *Arabidopsis* protoplast following a previous protocol [54]. While the empty vectors pC1300S-GFP and Ghd7:RFP were co-transformed as a control. The observation of GFP and RFP signals was performed on confocal laser scanning microscope (STELLARIS 8, Leica, Wetzlar, Germany).

### 4.4. Yeast Two-Hybrid Assay

The CDS of FtTT8 was amplified by specific primers (listed in Table S1) and recombined into a pGBKT7 (BD) vector, then the combination vector FtTT8-BD was co-transformed into the AH109 yeast strain with the PGADT7 (AD) vector using the LiAc-PEG-mediated transformation method. The combination of AD and BD was used as a negative control, while pGADT7-T and pGBKT7-p53 were used as positive controls. These yeast cells were cultured on a medium which lacked Leu and Trp (SD/-L/-T), followed by transferring several clones onto a medium lacking Leu, Trp, His, and Ade (SD/-L/-T/-H/-A), and SD/-L/-T/-H/-A with X-α-gal, respectively.

### 4.5. Transformation of Arabidopsis tt8 Mutant and Tobacco by FtTT8 Gene

The CDS of *FtTT8* was amplified by PCR using specific primers (Table S1) and recombined into the overexpression vector PMDC83, the constructed 35S:*FtTT8* vector was transformed into the *Arabidopsis tt8* mutant using the *Agrobacterium*-mediated floral dip method [16,54]. The T3 homozygous transgenic lines were used for further analysis. Meanwhile, the 35S:*FtTT8* transformed into tobacco (K326 cultivar with red flowers) by *Agrobacterium* through leaf disc transformation and the pigmentation of the transgenic tobacco was analyzed.

### 4.6. Detection of Anthocyanins and Proanthocyanidins (PAs)

The total anthocyanins of the fresh leaves of the WT and transgenic *Arabidopsis* plants were extracted following the protocol described by Park et al. [55]. Then, the extracted solutions were measured at absorption lengths of 530 nm and 657 nm using a UV9600 spectrophotometer (Beifen-Ruili, Beijing, China), and the total anthocyanin content was obtained by the formula Q_Anthocyanin_ = (A_530_ − 0.25 × A_657_)/M (Q represents the total anthocyanin content, M represents the sample weight). Each sample was processed with three biological replicates.

p-Dimethylaminocinnamaldehyde (DMACA) solution (0.1% *w*/*v* in methanol containing 1% (*v*/*v*) HCl) was used to visualize the PAs according to the method described by Abeynayake et al. [56]. Briefly, fresh samples (*Arabidopsis* seeds, leaves, and tobacco leaves and flowers) were soaked in GAA solution (ethyl alcohol: glacial acetic acid = 1:1 (*v*/*v*)) for full de-coloration first, then stained with 0.1% DMACA for 20 min with slow shaking. After staining, the samples were washed with 70% ethyl alcohol until the color was stable and were examined under the microscope. Meanwhile, the content of PAs was measured. In brief, firstly, standard of procyanidin B2 was accurately weighted and dissolved in methanol, which was then diluted 2-fold to produce appropriate concentration ranges, which were 7.81~125 μg/mL, to establish the calibration curve in order to quantify the PAs, while methanol was used as blank control. Samples were ground into fine power in liquid N_2_, then weighed at 5 mg into a 10 mL tube, respectively, 5 mL methanol was added, and ultrasonic extraction was performed for 20 min. After centrifugation, the filter residue was washed with methanol 3 times, and the washing liquids were combined with filtrates into a 10 mL measuring flask and diluted with methanol to volume and mixed well. Then, 1 mL of standard series solution and sample solution were collected, respectively, and then mixed with 6 mL of HCl-n-butanol (5:95, *v*/*v*) and 0.2 mL of NH_4_FeSO_4_ and heated in a boiling water bath for 40 min. They were then cooled to room temperature in ice water and the absorbance at the wavelength of 546 nm of the UV9600 spectrophotometer (Beifen-Ruili, Beijing, China) was measured. The PA content was obtained by calibration curve.

### 4.7. qRT-PCR Analysis

The expression levels of the structural genes on the pathways of anthocyanin and PA biosynthesis (*AtCHS*, *AtCHI*, *AtF3H*, *AtDFR*, *AtANS*, *AtUFGT* and *AtANR*) in wild-type (WT) and transgenic *Arabidopsis thaliana* seedlings and seeds, as well as *NtCHS*, *NtCHI*, *NtF3H*, *NtDFR*, *NtANS*, *NtUFGT*, *NtLAR*, and *NtANR* in WT and transgenic K326 tobacco were detected via qRT-PCR. QRT-PCR was performed using the C1000^TM^ thermal cycler coupled with a CFX96^TM^ detection module (Bio-Rad, Santa Clara, CA, USA) using the 2 × iQ^TM^ SYBR Green Super mix (Bio-Rad, Santa Clara, CA, USA). *AtActin* [57] and *NtActin* [58] were used as the internal controls. The expression levels of the genes were obtained by the 2^−ΔΔCt^ method with three technical replicates. The primers of these genes are listed in Table S1.

### 4.8. Yeast One-Hybrid Analysis

A 2000 bp promoter sequence of *AtUFGT* and *AtANR* was amplified by PCR and cloned into the pHIS2 vector, respectively, and the CDS of *FtTT8* was cloned into a pGADT7 (AD) vector. Then, the combination vector AD-FtTT8 was co-transformed into the AH109 yeast strain with vectors pHIS2-proAtUFGT and pHIS2-proAtANR, respectively. The combination of AD-p53 and pHIS2-p53 was used as a positive control, while the combinations of AD-p53 and pHIS2-proAtUFGT/pHIS2-proAtANR, AD-FtTT8, and pHIS2-p53 were negative controls. These yeast cells were cultured on a medium which lacks Leu and Trp (SD/-L/-T), then several clones were transferred to a medium lacking His, Leu, and Trp with 3AT (60 mM). The primers of these genes are listed in Table S1.

## 5. Conclusions

In the present study, FtTT8, a bHLH transcription factor, is located in the nucleus and exhibits transcriptional activation activity. Unlike TT8 from other plant species, which either promotes both anthocyanin and PA biosynthesis or inhibits the biosynthesis of these flavonoid components, FtTT8 suppresses anthocyanin biosynthesis but improves PA accumulation. In addition, FtTT8 may inhibit *UFGT* expression and activate *LAR* expression, respectively, by binding to G-boxes in the promoters of these two key genes that regulates anthocyanin and PA biosynthesis. This new insight enriches the roles of TT8 and sets the stage for further in-depth studies of the mechanism of TT8 regulation in flavonoid biosynthesis.

## Data Availability

Data are contained within the article and supplementary materials.

## Supplementary Materials

The following supporting information can be downloaded at: https://www.mdpi.com/article/10.3390/ijms242417368/s1.

## References

[1] Huda M.N., Lu S., Jahan T., Ding M., Jha R., Zhang K., Zhang W., Georgiev M.I., Park S.U., Zhou M. (2021). Treasure from garden: Bioactive compounds of buckwheat. Food Chem..

[2] Ohsako T., Ohnishi O. (2000). Intra-and interspecific phylogeny of wild *Fagopyrum* (Polygonaceae) species based on nucleotide sequences of noncoding regions in chloroplast DNA. Am. J. Bot..

[3] Tang Y., Ding M.Q., Tang Y.X., Wu Y.M., Shao J.R., Zhou M.L., Zhou M., Kreft I., Woo S.-H., Chrungoo N., Wieslander G. (2016). Germplasm resources of buckwheat in germplasm resources of buckwheat in China. Molecular Breeding and Nutritional Aspects of Buckwheat.

[4] Joshi D.C., Zhang K., Wang C., Chandora R., Khurshid M., Li J., He M., Georgiev M.I., Zhou M. (2020). Strategic enhancement of genetic gain for nutraceutical development in buckwheat: A genomics-driven perspective. Biotechnol. Adv..

[5] Christa K., Soral-Śmietana M. (2003). Buckwheat grains and buckwheat, products-nutritional and prophylactic value of their components—A review. Czech J. Food Sci..

[6] Zhang Z.L., Zhou M.L., Tang Y., Li F.L., Tang Y.X., Shao J.R., Xue W.T., Wu Y.M. (2012). Bioactive compounds in functional buckwheat food. Food Res. Int..

[7] Li H.Y., Lv Q.Y., Ma C., Qu J., Cai F., Deng J., Huang J., Ran P., Shi T., Chen Q. (2019). Metabolite profiling and transcriptome analyses provide insights into the flavonoid biosynthesis in the developing seed of Tartary buckwheat (*Fagopyrum tataricum*). J. Agric. Food Chem..

[8] Deng J., Dong F., Wu C., Zhao J., Li H.Y., Huang J., Shi T.X., Meng Z.Y., Cai F., Chen Q.F. (2021). Comparative metabolomics analysis between red- and white-flowered common buckwheat cultivars. Phyton-Int. J. Exp. Bot..

[9] Kreft S., Strukelj B., Gaberscik A., Kreft I. (2002). Rutin in buckwheat herbs grown at different UV-B radiation levels: Comparison of two UV spectrophotometric and an HPLC method. J. Exp. Bot..

[10] Wang H., Liu S., Cui Y., Wang Y., Guo Y., Wang X., Liu J., Piao C. (2021). Hepatoprotective effects of flavonoids from common buckwheat hulls in type 2 diabetic rats and HepG2 cells. Food Sci. Nutr..

[11] Javed H., Khan M.M., Ahmad A., Vaibhav K., Ahmad M.E., Khan A., Ashafaq M., Islam F., Siddiqui M.S., Safhi M.M. (2012). Rutin prevents cognitive impairments by ameliorating oxidative stress and neuroinflammation in rat model of sporadic dementia of Alzheimer type. Neuroscience.

[12] Zhong L., Lin Y., Wang C., Niu B., Xu Y., Zhao G., Zhao J. (2022). Chemical Profile, Antimicrobial and antioxidant activity assessment of the crude extract and its main flavonoids from Tartary buckwheat sprouts. Molecules.

[13] Koyama M., Nakamura C., Nakamura K. (2013). Changes in phenols contents from buckwheat sprouts during growth stage. J. Food Sci. Technol..

[14] Dong Y., Wang N., Wang S., Wang J., Peng W. (2023). A review: The nutrition components, active substances and flavonoid accumulation of Tartary buckwheat sprouts and innovative physical technology for seeds germinating. Front. Nutr..

[15] Kim S.J., Maeda T., Sarker M.Z., Takigawa S., Matsuura-Endo C., Yamauchi H., Mukasa Y., Saito K., Hashimoto N., Noda T. (2007). Identification of anthocyanins in the sprouts of buckwheat. J. Agric. Food Chem..

[16] Deng J., Wang L., Damaris R.N., Zhao J., Zhang L., Wang T., Yang C., Huang J., Shi T., Zhu L. (2023). Genome-wide identification of R2R3-MYB transcription factor family in Tartary buckwheat (*Fagopyrum tataricum*) identifies a member involved in anthocyanin biosynthesis. Agronomy.

[17] Li Z., Ahammed G.J. (2023). Hormonal regulation of anthocyanin biosynthesis for improved stress tolerance in plants. Plant Physiol. Biochem..

[18] Kaur S., Tiwari V., Kumari A., Chaudhary E., Sharma A., Ali U., Garg M. (2023). Protective and defensive role of anthocyanins under plant abiotic and biotic stresses: An emerging application in sustainable agriculture. J. Biotechnol..

[19] Šamec D., Karalija E., Šola I., Vujčić Bok V., Salopek-Sondi B. (2021). The role of polyphenols in abiotic stress response: The influence of molecular structure. Plants.

[20] Zhao P., Chu L., Wang K., Zhao B., Li Y., Yang K., Wan P. (2022). Analyses on the pigment composition of different seed coat colors in adzuki bean. Food Sci. Nutr..

[21] Vaughan S.P., Baker J.M., Primavesi L.F., Patil A., King R., Hassani-Pak K., Kulasekaran S., Coghill J., Ward J.L., Huttly A.K. (2022). Proanthocyanidin biosynthesis in the developing wheat seed coat investigated by chemical and RNA-Seq analysis. Plant Direct..

[22] Zhang Y., Qin Y., Li D., Wang W., Gao X., Hao C., Feng H., Wang Y., Li T. (2023). Fine mapping and cloning of a novel BrSCC1 gene for seed coat color in *Brassica rapa* L.. Theor. Appl. Genet..

[23] Wei J., Yang J., Jiang W., Pang Y. (2020). Stacking triple genes increased proanthocyanidins level in *Arabidopsis thaliana*. PLoS ONE.

[24] Shan X., Li Y., Yang S., Gao R., Zhou L., Bao T., Han T., Wang S., Gao X., Wang L. (2019). A functional homologue of *Arabidopsis* TTG1 from *Freesia* interacts with bHLH proteins to regulate anthocyanin and proanthocyanidin biosynthesis in both *Freesia hybrida* and *Arabidopsis thaliana*. Plant Physiol. Biochem..

[25] Kleindt C.K., Stracke R., Mehrtens F., Weisshaar B. (2010). Expression analysis of flavonoid biosynthesis genes during *Arabidopsis* thaliana silique and seed development with a primary focus on the proanthocyanidin biosynthetic pathway. BMC Res. Notes.

[26] Li G., Michaelis D.F., Huang J., Serek M., Gehl C. (2023). New insights into the genetic manipulation of the R2R3-MYB and *CHI* gene families on anthocyanin pigmentation in *Petunia hybrida*. Plant Physiol. Biochem..

[27] Zhang H., Koes R., Shang H., Fu Z., Wang L., Dong X., Zhang J., Passeri V., Li Y., Jiang H. (2019). Identification and functional analysis of three new anthocyanin R2R3-MYB genes in *Petunia*. Plant Direct..

[28] Chatham L.A., Juvik J.A. (2021). Linking anthocyanin diversity, hue, and genetics in purple corn. G3.

[29] Chatham L.A., Paulsmeyer M., Juvik J.A. (2019). Prospects for economical natural colorants: Insights from maize. Theor. Appl. Genet..

[30] Lim S.H., Kim D.H., Lee J.Y. (2022). RsTTG1, a WD40 Protein, Interacts with the bHLH Transcription Factor RsTT8 to regulate anthocyanin and proanthocyanidin biosynthesis in *Raphanus sativus*. Int. J. Mol. Sci..

[31] Liu W., Feng Y., Yu S., Fan Z., Li X., Li J., Yin H. (2021). The Flavonoid biosynthesis network in plants. Int. J. Mol. Sci..

[32] Xu W., Dubos C., Lepiniec L. (2015). Transcriptional control of flavonoid biosynthesis by MYB-bHLH-WDR complexes. Trends Plant Sci..

[33] Zhang L., Li X., Ma B., Gao Q., Du H., Han Y., Li Y., Cao Y., Qi M., Zhu Y. (2017). The Tartary buckwheat genome provides insights into rutin biosynthesis and abiotic stress tolerance. Mol. Plant..

[34] Bai Y.C., Li C.L., Zhang J.W., Li S.J., Luo X.P., Yao H.P., Chen H., Zhao H.X., Park S.U., Wu Q. (2014). Characterization of two tartary buckwheat R2R3-MYB transcription factors and their regulation of proanthocyanidin biosynthesis. Physiol. Plant.

[35] Wang L., Deng R., Bai Y., Wu H., Li C., Wu Q., Zhao H. (2022). Tartary buckwheat R2R3-MYB gene *FtMYB3* negatively regulates anthocyanin and proanthocyanidin biosynthesis. Int. J. Mol. Sci..

[36] Huang Y., Wu Q., Wang S., Shi J., Dong Q., Yao P., Shi G., Xu S., Deng R., Li C. (2019). FtMYB8 from Tartary buckwheat inhibits both anthocyanin/proanthocyanidin accumulation and marginal trichome initiation. BMC Plant Biol..

[37] Dong Q., Zhao H., Huang Y., Chen Y., Wan M., Zeng Z., Yao P., Li C., Wang X., Chen H. (2020). FtMYB18 acts as a negative regulator of anthocyanin/proanthocyanidin biosynthesis in Tartary buckwheat. Plant Mol. Biol..

[38] Wen C.H., Tsao N.W., Wang S.Y., Chu F.H. (2021). Color variation in young and senescent leaves of Formosan sweet gum (*Liquidambar formosana*) by the gene regulation of anthocyanidin biosynthesis. Physiol. Plant..

[39] Zhang D., Jiang C., Huang C., Wen D., Lu J., Chen S., Zhang T., Shi Y., Xue J., Ma W. (2019). The light-induced transcription factor FtMYB116 promotes accumulation of rutin in *Fagopyrum tataricum*. Plant Cell Environ..

[40] Qian W., Tan G., Liu H., He S., Gao Y., An C. (2007). Identification of a bHLH-type G-box binding factor and its regulation activity with G-box and Box I elements of the *PsCHS1* promoter. Plant Cell Rep..

[41] Xu W., Grain D., Le Gourrierec J., Harscoët E., Berger A., Jauvion V., Scagnelli A., Berger N., Bidzinski P., Kelemen Z. (2013). Regulation of flavonoid biosynthesis involves an unexpected complex transcriptional regulation of TT8 expression, in *Arabidopsis*. New Phytol..

[42] Ni J., Bai S., Zhao Y., Qian M., Tao R., Yin L., Gao L., Teng Y. (2019). Ethylene response factors Pp4ERF24 and Pp12ERF96 regulate blue light-induced anthocyanin biosynthesis in ‘Red Zaosu’ pear fruits by interacting with MYB114. Plant Mol. Biol..

[43] Ni J., Zhao Y., Tao R., Yin L., Gao L., Strid Å., Qian M., Li J., Li Y., Shen J. (2020). Ethylene mediates the branching of the jasmonate-induced flavonoid biosynthesis pathway by suppressing anthocyanin biosynthesis in red Chinese pear fruits. Plant Biotechnol. J..

[44] Deng J., Li J., Su M., Lin Z., Chen L., Yang P. (2021). A *bHLH* gene *NnTT8* of *Nelumbo nucifera* regulates anthocyanin biosynthesis. Plant Physiol. Biochem..

[45] Schaart J.G., Dubos C., Romero De La Fuente I., van Houwelingen A.M.M.L., de Vos R.C.H., Jonker H.H., Xu W., Routaboul J.M., Lepiniec L., Bovy A.G. (2013). Identification and characterization of MYB-bHLH-WD40 regulatory complexes controlling proanthocyanidin biosynthesis in strawberry (*Fragaria* × *ananassa*) fruits. New Phytol..

[46] Li P., Chen B., Zhang G., Chen L., Dong Q., Wen J., Mysore K.S., Zhao J. (2016). Regulation of anthocyanin and proanthocyanidin biosynthesis by *Medicago truncatula* bHLH transcription factor MtTT8. New Phytol..

[47] Hu R., Zhu M., Chen S., Li C., Zhang Q., Gao L., Liu X., Shen S., Fu F., Xu X. (2023). BnbHLH92a negatively regulates anthocyanin and proanthocyanidin biosynthesis in *Brassica napus*. Crop J..

[48] Zhao P., Li X., Jia J., Yuan G., Chen S., Qi D., Cheng L., Liu G. (2019). Corrigendum: bHLH92 from sheepgrass acts as a negative regulator of anthocyanin/proanthocyandin accumulation and influences seed dormancy. J. Exp. Bot..

[49] Gordân R., Shen N., Dror I., Zhou T., Horton J., Rohs R., Bulyk M.L. (2013). Genomic regions flanking E-box binding sites influence DNA binding specificity of bHLH transcription factors through DNA shape. Cell Rep..

[50] Ahmad A., Niwa Y., Goto S., Ogawa T., Shimizu M., Suzuki A., Kobayashi K., Kobayashi H. (2015). bHLH106 Integrates Functions of Multiple Genes through Their G-Box to Confer Salt Tolerance on *Arabidopsis*. PLoS ONE.

[51] Zhao J., Li H., Huang J., Shi T., Meng Z., Chen Q., Deng J. (2021). Genome-wide analysis of *BBX* gene family in Tartary buckwheat (*Fagopyrum tataricum*). PeerJ.

[52] Kumar S., Stecher G., Tamura K. (2016). MEGA7: Molecular evolutionary genetics analysis version 7.0 for bigger datasets. Mol. Biol. Evol..

[53] Chen C., Chen H., Zhang Y., Thomas H.R., Frank M.H., He Y., Xia R. (2020). TBtools: TBtools: An Integrative Toolkit Developed for Interactive Analyses of Big Biological Data. Mol. Plant..

[54] Xiong L., Li C., Li H., Lyu X., Zhao T., Liu J., Zuo Z., Liu B. (2019). A transient Expression System in Soybean Mesophyll Protoplasts Reveals the Formation of Cytoplasmic GmCRY1 Photobody-Like Structures. Sci. China Life Sci..

[55] Park N.I., Li X., Suzuki T., Kim S.J., Woo S.H., Park C.H., Park S.U. (2011). Differential Expression of Anthocyanin Biosynthetic Genes and Anthocyanin Accumulation in Tartary Buckwheat Cultivars ‘Hokkai t8’ and ‘Hokkai t10’. J. Agric. Food Chem..

[56] Abeynayake S.W., Panter S., Chapman R., Webster T., Rochfort S., Mouradov A., Spangenberg G. (2012). Biosynthesis of proanthocyanidins in white clover flowers: Cross talk within the flavonoid pathway. Plant Physiol..

[57] An X.H., Tian Y., Chen K.Q., Liu X.J., Liu D.D., Xie X.B., Cheng C.G., Cong P.H., Hao Y.J. (2015). MdMYB9 and MdMYB11 are Involved In the regulation of the JA-Induced Biosynthesis of Anthocyanin and Proanthocyanidin in Apples. Plant Cell Physiol..

[58] Zhao R., Song X., Yang N., Chen L., Xiang L., Liu X.Q., Zhao K. (2020). Expression of the subgroup IIIf bHLH transcription factor CpbHLH1 from *Chimonanthus praecox* (L.) in transgenic model plants inhibits anthocyanin accumulation. Plant Cell Rep..

